# Supplementary material for: Validity and reliability of the Serbian COVID Stress Scales
Source: PLoS One. 2021 Oct 27;16(10):e0259062. doi: 10.1371/journal.pone.0259062 (PMC8550439; doi:10.1371/journal.pone.0259062)
Supplement: S1 File — (DOC) [file pone.0259062.s001.doc]

**Validity and reliability of the Serbian COVID Stress Scales**

Supporting information

**S1 Appendix.** Detailed description of the COVID Stress Scales translation and modifications

**S1 Table.** Nuances and adjustments of the COVID Stress Scale translation to Serbian language

**S1 Appendix.** Detailed description of the COVID Stress Scales translation and modifications

We observed that the original scale used verbs in present simple passive, which is not a common tense in Serbian language. For this reason, the Serbian translation used present simple tense throughout the questionnaire. Moreover, instead of using present continuous tense, we used future tense in all items as it is more fitting for Serbian language. There were several modifications to the manner in which item content was expressed (Supplemental Table S1). For example, regarding item 10, it is not common in Serbia to buy over-the-counter medications in grocery stores, but rather in pharmacies or a drugstore. Further, for item 26 the term "popped into my mind" had to be changed to "run through my head" as that is an appropriate expression to communicate the same meaning in Serbian. Also, in item 30 instead of "bad dreams", we used "nightmares" as a more appropriate term. There is no single word in Serbian that would have the exact meaning to the word “reassurance” in the context of the item 35 and to explain the term, we would have to use a descriptive expression of feelings and actions; therefore, the approximation in Serbian is the word "support". In item 34 we used the word “myself” instead of “my body”, as in Serbian language, “myself” refers to a person as an object that performs certain action (general and physical form) and, therefore, this is the shortest word to substitute the word “body”.

**S1 Table**. Nuances and adjustments of the COVID Stress Scale translation to Serbian language

| No. | Original wording of items | Wording in Serbian |
| --- | --- | --- |
| 1 | I am worried about catching the virus | I worry about getting the virus |
| 2 | I am worried that I can’t keep my family safe from the virus | I worry that I cannot protect my family against the virus |
| 3 | I am worried that our healthcare system won’t be able to protect my loved ones | I worry that our healthcare system won’t be able to protect persons who I love |
| 4 | I am worried our healthcare system is unable to keep me safe from the virus | I worry that out health care system is not be able to protect me from the virus |
| 5 | I am worried that basic hygiene is not enough to keep me safe from the virus | I worry that basic hygiene (e.g. hand washing) is not enough to protect me from the virus |
| 6 | I am worried that social distancing is not enough to keep me safe from the virus | I worry that social distancing is not enough to protect me from the virus |
| 7 | I am worried about grocery stores running  out of food | I worry that grocery stores will run out of food |
| 8 | I am worried that grocery stores will  close down | I worry that grocery stores will close down |
| 9 | I am worried about grocery stores running out of cleaning or disinfectant supplies | I worry that stores will run out of cleaning and disinfection supplies |
| 10 | I am worried about grocery stores running out of cold or flu remedies | I worry that pharmacies/grocery stores will run out of cold or flu remedies |
| 11 | I am worried about grocery stores running out of water | I worry that stores will run out without water |
| 12 | I am worried about pharmacies running out of prescription medicines | I worry that pharmacies are left without prescription medications |
| 13 | I am worried that foreigners are spreading the virus in my country | I worry that foreigners spread the virus in my country |
| 14 | If I went to a restaurant that specialized in foreign foods, I’d be worried about catching the virus | If I went to a restaurant that specialized in foreign cuisine, I’d be worried about getting the virus |
| 15 | I am worried about coming into contact with foreigners because they might have the virus | I worry about being in contact with foreigners because they might have the virus |
| 16 | If I met a person from a foreign country, I’d be worried that they might have the virus | If I met a foreign person I would be worried that they are infected with the virus |
| 17 | If I was in an elevator with a group of foreigners, I’d be worried that they’re infected with the virus | If I found myself in an elevator with a group of foreigners I would worry that they might be infected with the virus. |
| 18 | I am worried that foreigners are spreading the virus because they’re not as clean as we are | I worry that foreigners spread the virus because they are not as clean as we are |
| 19 | I am worried that if I touched something in a public space, I would catch the virus | I worry that if I touch something in public space (e.g. rails, door knob) I would contract the virus |
| 20 | I am worried that if someone coughed or sneezed near me, I would catch the virus | I worry that if someone is coughing or sneezing close to me I would contract the virus |
| 21 | I am worried that people around me will infect me with the virus | I worry about contracting the virus from people who are around me |
| 22 | I am worried about taking change in  cash transactions | I worry about taking change when I pay in cash |
| 23 | I am worried that I might catch the virus from handling money or using a debit machine | I worry I might contract the virus by handling money or using the ATM |
| 24 | I am worried that my mail has been contaminated by mail handlers | I worry that mailman contaminates my mail |
| 25 | I had trouble concentrating because I kept thinking about the virus | I had trouble concentrating because I think about the virus |
| 26 | Disturbing mental images about the virus popped into my mind against my will | Disturbing images about the virus run through my head against my will |
| 27 | I had trouble sleeping because I worried about the virus | I slept poorly because I worried about the virus |
| 28 | I thought about the virus when I  didn’t mean to | I was thinking about the virus even when I did not want to think about it. |
| 29 | Reminders of the virus caused me to have physical reactions, such as sweating or a pounding heart | Things that remind me of the virus cause me to have physical reactions such as sweating or increased heart beating |
| 30 | I had bad dreams about the virus | I had nightmares about the virus |
| 31 | Searched the Internet for treatments for COVID-19 | I searched on the Internet about treatments for COVID-19 |
| 32 | Asked health professionals (e.g., doctors or pharmacists) for advice about COVID-19 | I asked health care workers (e.g. doctors, pharmacists) for advice about COVID-19 |
| 33 | Checked YouTube videos about COVID-19 | I watched YouTube videos about COVID-19 |
| 34 | Checked your own body for signs of infection (e.g., taking your temperature) | I searched myself looking for signs of infection (e.g. measure my body temperature) |
| 35 | Sought reassurance from friends or family  about COVID-19 | I sought support from friends or family because of COVID-19 |
| 36 | Checked social media posts concerning COVID-19 | I was checking the social media posts to see what was written about COVID-19 |
